# Does smoking cessation reduce other substance use, psychiatric symptoms, and pain symptoms? Results from an emulated hypothetical randomized trial of US veterans

**DOI:** 10.1371/journal.pone.0298576

**Published:** 2024-07-03

**Authors:** Kaoon (Francois) Ban, Erin Rogers, Maria Khan, Joy Scheidell, Dyanna Charles, Kendall J. Bryant, Amy C. Justice, R. Scott Braithwaite, Ellen C. Caniglia

**Affiliations:** 1 Department of Population Health, NYU Grossman School of Medicine, New York, New York, United States of America; 2 National Institutes of Health, Bethesda, Maryland, United States of America; 3 Yale School of Medicine and Public Health, New Haven, Connecticut, United States of America; 4 VA Connecticut Healthcare System, West Haven, Connecticut, United States of America; 5 Department of Biostatistics, Epidemiology and Informatics, University of Pennsylvania Perelman School of Medicine, Philadelphia, Pennsylvania, United States of America; University of Toronto, CANADA

## Abstract

**Background:**

Quitting smoking may lead to improvement in substance use, psychiatric symptoms, and pain, especially among high-risk populations who are more likely to experience comorbid conditions. However, causal inferences regarding smoking cessation and its subsequent benefits have been limited.

**Methods:**

We emulated a hypothetical open-label randomized control trial of smoking cessation using longitudinal observational data of HIV-positive and HIV-negative US veterans from 2003–2015 in the Veterans Aging Cohort Study. We followed individuals from the first time they self-reported current cigarette smoking (baseline). We categorized participants as quitters or non-quitters at the first follow-up visit (approximately 1 year after baseline). Using inverse probability weighting to adjust for confounding and selection bias, we estimated odds ratios for improvement of co-occurring conditions (unhealthy alcohol use, cannabis use, illicit opioid use, cocaine use, depressive symptoms, anxiety symptoms, and pain symptoms) at second follow-up (approximately 2 years after baseline) for those who quit smoking compared to those who did not, among individuals who had the condition at baseline.

**Results:**

Of 4,165 eligible individuals (i.e., current smokers at baseline), 419 reported no current smoking and 2,330 reported current smoking at the first follow-up. Adjusted odds ratios (95% confidence intervals) for associations between quitting smoking and improvement of each condition at second follow-up were: 2.10 (1.01, 4.35) for unhealthy alcohol use, 1.75 (1.00, 3.06) for cannabis use, 1.10 (0.58, 2.08) for illicit opioid use, and 2.25 (1.20, 4.24) for cocaine use, 0.78 (0.44, 1.38) for depressive symptoms, 0.93 (0.58, 1.49) for anxiety symptoms, and 1.31 (0.84, 2.06) for pain symptoms.

**Conclusions:**

While a causal interpretation of our findings may not be warranted, we found evidence for decreased substance use among veterans who quit cigarette smoking but none for the resolution of psychiatric conditions or pain symptoms. Findings suggest the need for additional resources combined with smoking cessation to reduce psychiatric and pain symptoms for high-risk populations.

## Introduction

Cigarette smoking accounts for more than 25% of deaths in the United States (US) and is the leading cause of morbidity and preventable mortality in the US [1]. Although the prevalence of cigarette smoking among adults has decreased substantially in the past 15 years, 11.5% of adults are still currently smoking cigarettes [1]. Cigarette smoking does not happen in isolation, but rather commonly co-occurs with substance use [2–5], psychiatric conditions including depression [4, 6, 7] and anxiety [7–9], and chronic pain symptoms [10]. While these tobacco-related co-occurring factors are prevalent on their own, data from the National Epidemiologic Survey and Related Conditions suggests that the co-use of tobacco and alcohol in the past year was as high as 21.7% among U.S. adults [3]. Alongside alcohol use, marijuana is the next most commonly used illicit substance and has been used commonly with tobacco [11]. A national survey of U.S. adults found that prevalence of any illicit drug use (e.g., cannabis, cocaine, opioids, hallucinogens, and non-cocaine stimulants) was more common among current cigarette smokers than former or never smokers [12]. Further, lifetime smoking rates are higher in patients diagnosed with mental health outcomes than those without [13], and cigarette smoking has continued to be prevalent among patients experiencing pain [14].

Additionally, high risk populations, such as people living with HIV (PLHIV) and military veterans, have higher co-morbidity of cigarette smoking, substance use, psychiatric conditions, and pain, and they may also be less likely to access treatment [15–18]. For instance, prevalence of current cigarette smoking is observed to be as high as 33.6% among HIV-infected individuals [18] and 12.9% among veterans enrolled in care [19] while prevalence of illicit drug use are as high as 40% among HIV-infected populations [15]. Further, prevalence of life-time and past-year probable alcohol use disorder was as high as 42.2% and 14.8% respectively, among US veterans [20]. Given the prevalence of these conditions among PLHIV and among veterans, we sought to investigate if reducing cigarette smoking may lead to subsequent improvements in other conditions, which could inform treatment strategies for smoking cessation.

Relationships between smoking cessation and substance use have been observed in several substance use treatment settings. In a meta-analysis of 19 RCTs, individuals undergoing substance use treatment who were randomized to receive additional smoking cessation interventions had greater long-term abstinence from alcohol and illicit substance use compared to individuals not randomized to smoking cessation treatment [21]. However, the authors reported that the included studies had low quality scores due to several methodological limitations such as unbalanced study design, inconsistent measurement of smoking abstinence, and small sample size. In a separate systematic review of 24 observational and randomized studies examining the impact of quitting smoking or smoking cessation interventions on substance abuse, 11 studies found a reduction in substance use with smoking cessation, 12 studies found either a positive or null impact of smoking cessation on substance use, and one study reported mixed negative and null impact of smoking cessation on substance use [22]. However, less than half these studies were RCTs, and the ambiguous nature of the effects of smoking cessation on subsequent reduction in substance use calls for additional rigorous statistical approach.

While many studies have explored associations of smoking with psychiatric disorders, their scope of causal inference was limited. In a recent systemic review of randomized clinical trial (RCT) data and longitudinal observational studies of smoking cessation studies, smoking cessation was associated with improvement in mental health symptoms including depression and anxiety compared to continued smoking [23]. The effect sizes for each psychiatric outcome was small to moderate. However, the authors concluded low certainty in the estimates of the impact of smoking cessation on depression and anxiety as all studies included in the meta-analysis were at risk for time-varying confounding biases. Additionally, more than 50% of the studies included were individuals from the general population and only about 22% of the studies included individuals with psychiatric disorders.

Various pain symptoms have been explored in the context of smoking cessation, and literature suggests a potential bidirectional relationship [24, 25]. In a secondary analysis of an RCT for Veterans who smoked cigarettes and were receiving Veterans Health Administration (VHA) mental health care, there was a significant association between short-term smoking abstinence and a reduction in pain levels at six months [26]. However, these results were not sustained at 12 months, and the study controlled for few sociodemographic and psychosocial variables. Thus, the direction and nature of the relationship between cigarette smoking and pain remains unclear.

While prior studies have identified associations between smoking cessation or smoking cessation interventions and improvement of substance use, psychiatric conditions, and pain symptoms, their scope of causal inference was limited. Many were secondary analyses of RCTs that did not control for additional co-occurring conditions other than sociodemographic variables. Most prior studies had limited information about timing of events so reverse causation cannot not be ruled out. Accordingly, we sought to investigate strength of causal inferences between smoking cessation and improvements in substance use, psychiatric symptoms, and pain symptoms by emulating a hypothetical randomized controlled trial.

Using observational data, we perform distinct analyses for different substances including alcohol, cannabis, opioid, and cocaine as well as depressive, anxiety and pain symptoms. We first specify each component of the hypothetical trial [27, 28]–the target trial–including the eligibility criteria, treatment strategies, outcomes, and the start and end of follow-up. We then attempt to emulate each component using observational data. The target trial framework helps to avoid selection and confounding biases that are common weaknesses of observational studies and to identify potential limitations of the analytic plan.

## Materials and methods

### Study population

The Veterans Aging Cohort Study (VACS) includes US veterans receiving healthcare in nine VHA medical centers located in Atlanta, Baltimore, New York, Houston, Los Angeles, Pittsburgh, and Washington, DC. Composed of clinical and survey data, the VACS includes approximately 3,500 veterans living with HIV and 3,500 HIV-uninfected controls, frequency matched by age, race, gender, and site [29]. Study enrollment began in 2002 and surveys were administered approximately annually from 2003 to 2015 in Atlanta, New York, Houston, Los Angeles, and Pittsburgh and from 2004 to 2015 in Baltimore and Washington, DC. Surveys included demographic and clinical information on HIV risk factors, pain, alcohol use, anxiety symptoms, depressive symptoms, and other substance use. Institutional review boards at each participating VHA center, New York University, and Yale University approved all study activities.

### Current cigarette smoking

Smoking status was ascertained at each survey [30]. The National Survey on Drug Use and Health (NSDUH), a national population survey, defines ‘current smoker’ as having smoked within the past 30 days [31]. For our study, individuals who reported that they “now smoke cigarettes (e.g., within the past week)” or quit smoking within the last four weeks were coded as “Current” smokers. Individuals who reported to have ever smoked cigarettes for “as long as a year” and quit smoking more than four weeks ago were coded as “Former.” Individuals who reported that they did not currently smoke or did not ever smoke for as long as a year were coded as “Never.”

### Past-year unhealthy alcohol use

Alcohol use was measured using the alcohol use disorder identification test (AUDIT), a 10-item questionnaire designed to detect hazardous or harmful drinking [32]. The AUDIT assesses past-year alcohol consumption, dependence symptoms, and consequences of use like guilt, alcohol-related injury, and others’ concern about one’s use. Each item is scored from 0–4 for a total score of 0–40. Missing AUDIT score data were characterized using available AUDIT items consistent with previous analyses [33]. Consistent with World Health Organization guidelines [34], we considered AUDIT scores ≥8 as unhealthy alcohol use.

### Other past-year substance use

At each survey, individuals were asked about their substance use over the past-year, including use of crack/cocaine, cannabis, other stimulants (e.g., amphetamine), and illicit opioid use. We defined self-reported illicit opioid use as non-medical use of prescription opioids (e.g., Oxycontin, Vicodin, Percocet) or heroin [33, 35].

### Current depressive and anxiety symptoms

At each survey, current (past two weeks) depressive symptoms were measured using the Patient Health Questionnaire (PHQ-9), a nine-item screening instrument that assesses the frequency of experiencing depression-related problems [36]. Individuals scoring 10 or more on the PHQ-9 were classified as currently having depressive symptoms and individuals scoring 9 or less were classified as having no depressive symptoms. Current anxiety symptoms were assessed by a single survey item which asked if participants had “felt nervous or anxious” in the past four weeks, and if applicable, the degree to which they were bothered on a four-point Likert scale. People who endorsed feeling nervous or anxious and who reported the symptoms “doesn’t bother me,” “bothers me a little,” “bothers me,” or “bothers me a lot” were coded as having anxiety symptoms [37].

### Current pain symptoms

To ascertain bodily pain, a single question derived from the 12-Item Short-Form Health Survey asked participants: “During the last month, how much has pain interfered with your normal work (including work outside and inside the home)?” [38]. We classified individuals who answered “moderately,” “quite a bit,” or “extremely” as having moderate or severe pain interference and “not at all” and “a little bit” as having little to no pain interference [38].

### Eligibility, treatment strategies, and start of follow-up

In our analysis, eligible individuals were those who indicated current smoking during at least one of the surveys. Baseline (the start of follow-up) was defined as the first survey at which the individual indicated current smoking. To emulate a target trial using observational data, we compared two ‘treatment’ strategies: 1) participants who stopped smoking at the first follow-up visit post baseline (approximately one year after their baseline visit) and 2) participants who continued smoking at the first follow-up visit post baseline. Participants who had a missing smoking status at the first follow-up visit or did not attend the first follow-up visit were classified as having an unknown smoking status.

### Outcome definitions and end of follow-up

The primary outcomes were: 1) improvement of depressive symptoms (defined as PHQ-9≤9), 2) resolution of anxiety symptoms (answering “I do not have this symptom” to the same anxiety question), 3) improvement of moderate or severe pain (answering “not at all” or “a little bit” to the same pain question, 4) reduction of alcohol use (AUDIT score <8), and 5) discontinuation of reporting cannabis, illicit opioids, and cocaine (no use in the past year) at the second follow-up (approximately two years after baseline) among participants who had that condition during completion of the baseline survey. Individuals were followed from baseline (e.g. the first survey in which they reported current smoking status) until second follow-up post baseline, or until the administrative end of follow-up, death, or censoring (defined as having an unknown smoking status at first follow-up post baseline).

### Statistical analyses

We fit separate logistic regression models to estimate odds ratios (ORs) for resolution of each outcome at second follow-up comparing participants who quit smoking to those who did not quit at first follow-up. For each of the seven outcomes, the analysis was restricted to individuals who had that condition at baseline. For example, specifically for the unhealthy alcohol use outcome, persons who indicated cigarette smoking *and* unhealthy alcohol use (AUDIT scores ≥8) at baseline were included in the analysis and the outcome was reduction in unhealthy alcohol use (AUDIT score <8) at the second follow-up.

Inverse probability weights were estimated to adjust for potential selection bias induced by censoring individuals who did not have smoking status measured at first follow-up [39]. We fit a logistic regression model to predict missingness of smoking status at first follow-up, conditional on the following baseline variables: HIV status, race, age, education, income, smoking status, alcohol, pain, depressive, and anxiety symptoms, and past-year cannabis, cocaine, other stimulants, and illicit opioid use. These weights were used to account for measured differences between those who did and did not have cigarette smoking status measured at the first follow-up.

**Fig 1:**
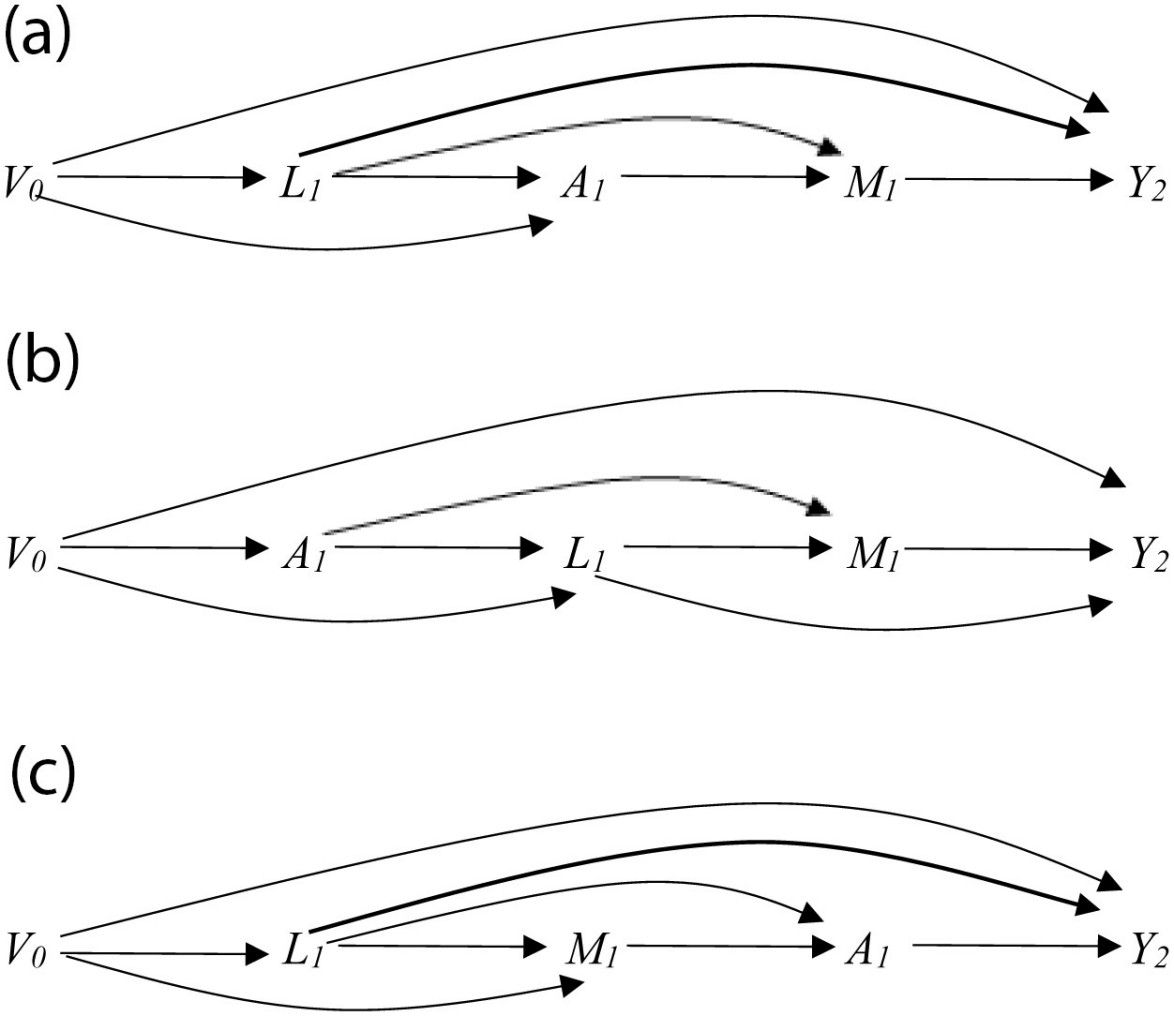
Causal directed acyclic graph (DAG) for estimating the effect of smoking cessation (*A*) on co-occurring conditions (*Y*) under various assumptions about the causal structure of the data. 1A depicts assumptions for the adjusted models with following temporal ordering of variables: *L*_*1*_, *A*_*1*_, *M*_*1*_. *Some* potential time-varying covariates (*L*_*1*_) measured at the first follow-up visit and *V*_*0*_ are *included* for the weights. 1B depicts assumptions for the adjusted models with following temporal ordering of variables: *A*_*1*_, *L*_*1*_, *M*_*1*_. *All* potential time-varying covariates measures at the first follow-up visit are *excluded* from the weight model. Only *V*_*0*_ included for the weights. 1C depicts assumptions for the adjusted models with following temporal ordering of variables: *L*_*1*_, *M*_*1*_, *A*_*1*_. *All* potential time-varying covariates measures at the first follow-up visit and *V*_*0*_ are *included* for the weights. ***V***_***0***_ Baseline covariates (e.g., HIV status, race, age, education, income), past year unhealthy alcohol use, cannabis, cocaine, other stimulants at time 0, and current smoking, depression, anxiety, and pain measured at time 0. ***L***_***1***_ Past year unhealthy alcohol use, cannabis, cocaine, other stimulants and opioids measured at first follow-up visit. ***A***_***1***_ Current cigarette smoking measured at first follow-up visit. ***M***_***1***_ Current depression, anxiety, and pain measured at first follow-up visit. ***Y***_***2***_ Outcome measured at second follow-up visit (for all outcomes).

To account for confounding in our primary analyses (e.g. any differences between those who did and did not quit smoking), we fit a logistic regression model for smoking status at first follow-up, conditional on having smoking status measured at first follow-up, the baseline variables mentioned above, and the following time-varying covariates measured at first follow-up: past-year unhealthy alcohol use, cannabis, illicit opioids, cocaine, and other stimulants. Based on the timeframe of how these variables were measured (participants were asked about use over the past year) compared with how the smoking variable was measured (participants were asked about current smoking), we assumed changes in these time-varying covariates occurred prior to or at the same time as changes in first follow-up smoking status, rather than occurring after changes in first follow-up smoking status (Fig 1a). We did not include time-varying covariates of depressive, anxiety, and pain symptoms measured at the first follow-up in the primary analysis because we could not assume changes in these covariates occurred prior to or at the same time as changes in smoking status (because participants were asked about more recent depressive, anxiety, and pain symptoms).

All uncensored individuals (those with cigarette smoking status measured at the first follow-up) received a weight that was inversely proportional to the product of their conditional probability of having smoking status measured and having the smoking status that the individual reported. The weights were stabilized and truncated at the 99th percentile. Logistic regression models to estimate associations between cessation of cigarette smoking and each outcome were then fit in the pseudo-population created by the inverse probability weights. Analyses were conducted overall and separately by HIV status to examine any differences in the impact of smoking cessation on the outcomes between PLHIV and HIV-uninfected veterans. Assuming no model misspecification, the inverse probability weights create a pseudo-population where selection bias and confounding by the measured covariates no longer exist [40].

As the exact time of smoking cessation is unknown between baseline and the next follow-up visit, confounding adjustment becomes challenging as it becomes unclear whether changes in co-occurring conditions between baseline and the next follow-up are causes or consequences of smoking cessation. Therefore, we performed the following sensitivity analyses: 1) assumed changes in smoking status between baseline and the first follow-up occurred *prior to* changes in cannabis, opioid, cocaine, and other stimulant use at first follow-up and *excluded* these variables to calculate the weights (Fig 1b), and 2) assumed changes in smoking status occurred *after* changes in depressive, anxiety, and pain symptoms, and included these variables to calculate the weights (Fig 1c).

We also calculated E-values to estimate the strength of unmeasured confounding needed to explain away the association between quitting smoking and each outcome of interest [41]. The E-value for the point estimate is defined as the minimum strength of association that an unmeasured confounder needs with the treatment and outcome, conditional on measured covariates, to fully explain away the association. The lower bound 95% confidence interval (CI) of the E-value is the minimum strength of association that an unmeasured confounder needs with both the exposure and outcome to shift the CI to contain the null value.

## Results

Of the 4,165 individuals who identified as people who smoke at some point during the VACS follow-up and were included in the analysis, 96.0% were male, 66.1% were African-American, 91.5% received at least a high school education, 53.0% had an annual household income of less than $12,000, and average age was 50 years old. Co-occurring conditions were fairly common with the most frequently reported outcome being moderate or severe pain symptoms (42.7%) and anxiety symptoms (48.4%), (Table 1).

**Table 1:** Baseline characteristics of individuals, overall and by current smoking status at the next survey, VACS.

Baseline characteristic	All individuals	Quit smoking at next survey	Current smokers at next survey	No smoking measure at next survey
Number (%)	n = 4165	n = 419	n = 2330	n = 1416
HIV status				
Positive	2153 (51.7)	254 (60.6)	1299 (55.8)	600 (42.4)
Negative	2012 (48.3)	165 (49.4)	1031 (44.3)	816 (57.6)
Race				
African-American	2755 (66.1)	289 (69.0)	1640 (70.4)	826 (58.3)
Other	1410 (33.9)	130 (31.0)	690 (29.6)	590 (41.7)
Biological Sex				
Male	3999 (96.0)	403 (96.2)	2229 (95.7)	1367 (96.5)
Female	166 (3.99)	16 (3.82)	101 (4.33)	49 (3.46)
Age				
Mean (SD)	49.6 (8.09)	49.3 (8.94)	49.4 (7.27)	49.9 (9.05)
Highest educational attainment				
Less than high school	308 (7.39)	25 (5.97)	188 (8.07)	95 (6.71)
High school or more	3809 (91.5)	387 (92.4)	2115 (90.8)	1307 (92.3)
Missing	48 (1.15)	7 (1.67)	27 (1.16)	14 (0.99)
Annual household income				
<$12,000	2207 (53.0)	197 (47.0)	1282 (55.0)	728 (51.4)
≥12,000	1819 (43.7)	204 (48.7)	969 (41.6)	646 (45.6)
Missing	139 (3.3)	18 (4.30)	79 (3.39)	42 (2.97)
Current (past-year) unhealthy alcohol use				
Yes	746 (17.9)	70 (16.7)	491 (21.1)	185 (13.1)
No	2642 (63.4)	310 (74.0)	1597 (68.5)	735 (51.9)
Missing	777 (18.7)	39 (9.31)	242 (10.4)	496 (35.0)
Current (past-year) cannabis use				
Yes	1198 (28.8)	108 (25.8)	666 (28.6)	424 (29.4)
No	2871 (68.9)	301 (71.8)	1600 (68.7)	970 (68.5)
Missing	96 (2.30)	10 (2.39)	64 (2.75)	22 (1.55)
Current (past-year) illicit opioid use				
Yes	847 (20.3)	85 (20.3)	468 (20.1)	294 (20.8)
No	3137 (75.3)	311 (74.2)	1742 (74.8)	1084 (76.6)
Missing	181 (2.68)	23 (5.49)	120 (5.15)	38 (2.68)
Current (past-year) cocaine use				
Yes	1057 (25.4)	88 (21.0)	625 (26.8)	344 (24.3)
No	2988 (71.7)	316 (75.4)	1631 (70.0)	1041 (73.5)
Missing	120 (2.88)	15 (3.58)	74 (3.18)	31 (2.19)
Current (past-year) other stimulant use				
Yes	188 (4.51)	24 (5.73)	85 (3.65)	79 (5.58)
No	3842 (92.2)	380 (90.7)	2164 (92.9)	1298 (91.7)
Missing	145 (3.24)	15 (3.58)	81 (3.48)	39 (2.75)
Current (past 2 weeks) depressive symptoms				
Yes	1087 (26.1)	93 (22.2)	609 (26.1)	385 (27.2)
No	3011 (72.3)	320 (76.4)	1685 (72.3)	1006 (71.1)
Missing	67 (1.61)	6 (1.43)	36 (1.55)	25 (1.77)
Current (past 4 weeks) anxiety symptoms				
Yes	2016 (48.4)	170 (40.6)	1093 (46.9)	753 (53.2)
No	1741 (41.8)	179 (42.7)	1030 (44.2)	532 (37.6)
Missing	408 (9.80)	70 (16.7)	207 (8.88)	131 (9.25)
Current (past month) moderate or severe pain symptoms				
Yes	1780 (42.7)	152 (36.3)	979 (42.0)	649 (45.8)
No	2308 (55.4)	255 (60.9)	1305 (56.0)	748 (52.8)
Missing	77 (1.85)	12 (2.86)	46 (1.97)	19 (1.34)

At the first follow-up visit, 419 (10.1%) individuals quit smoking, 2,330 (55.9%) did not quit smoking, and 1,416 (34.0%) did not have data regarding smoking status as they did not participate in that follow-up. Individuals who quit smoking at the follow-up visit were more likely to be living with HIV, were less likely to have an annual household income less than $12,000, and less likely to have had anxiety symptoms at baseline than individuals who did not quit smoking at the follow-up visit (Table 1). Individuals without a smoking measure at the follow-up visit were less likely to be African-American, more likely to have had anxiety symptoms at baseline, and less likely to have used unhealthy alcohol at baseline than individuals with a smoking measure. Those who did not have a smoking measure at the follow-up visit did not appear to differ on any other baseline characteristics. Of the 2,749 with a defined smoking status at first follow-up after baseline, 1,977 (71.9%) had follow-up at second follow-up (66.6% of those who quit smoking and 72.0% of those who did not quit smoking at first follow-up).

### Alcohol and other substances outcomes

Among individuals who reported past-year unhealthy alcohol use at baseline, the AOR for reducing drinking was 2.13 (1.03, 4.39) at second follow-up comparing quitting smoking versus continued smoking. Among individuals reporting cannabis use in the past-year at baseline, the AOR for no longer using cannabis at second follow-up was 1.67 (0.94, 2.97) comparing quitting smoking versus continued smoking. Among individuals reporting past-year illicit opioid use at baseline, the AOR for no longer using illicit opioids at second follow-up was 1.09 (0.57, 2.07) comparing quitting smoking versus continued smoking. Among individuals reporting past-year cocaine use at baseline, the AOR for no longer using cocaine at second follow-up was 2.12 (1.11, 4.03) comparing quitting smoking versus continued smoking.

### Depressive, anxiety, and pain symptom outcomes

Among individuals with depressive symptoms at baseline, the AOR for improvement of depressive symptoms was 0.74 (0.41, 1.34) at second follow-up comparing quitting smoking versus continued smoking. Among individuals with anxiety symptoms at baseline, the adjusted odds ratio or AOR (95% CI) for no longer having anxiety symptoms were 0.98 (0.60, 1.60) at second follow-up, comparing quitting smoking versus continued smoking (Table 2). Among individuals with moderate or severe pain symptoms at baseline, the AOR for improvement of pain symptoms were 1.24 (0.78, 1.95) at second follow-up comparing quitting smoking versus continued smoking.

**Table 2:** Odd ratios for each condition improving or resolving comparing quitters to non-quitters at first follow-up.

Condition improves	Analysis	Odds ratios (95% CIs) at second follow-up
Past-year unhealthy alcohol use	Unadjusted	3.09 (1.59, 5.99)
	Adjusted*	2.13 (1.03, 4.39)
Past-year cannabis use	Unadjusted	2.08 (1.25, 3.47)
	Adjusted*	1.67 (0.94, 2.97)
Past-year illicit opioid use	Unadjusted	1.07 (0.61, 1.89)
	Adjusted*	1.09 (0.57, 2.07)
Past-year cocaine use	Unadjusted	2.93 (1.64, 5.24)
	Adjusted*	2.12 (1.11, 4.03)
Current depressive symptoms	Unadjusted	0.91 (0.55, 1.52)
	Adjusted*	0.74 (0.41, 1.34)
Current anxiety symptoms	Unadjusted	1.28 (0.83, 2.00)
	Adjusted*	0.98 (0.60, 1.60)
Current moderate or severe pain symptoms	Unadjusted	1.44 (0.95, 2.18)
	Adjusted*	1.24 (0.78, 1.95)

*Adjusted for HIV status, race, age, education, income, baseline conditions (AUDIT score, depressive symptoms, anxiety symptoms, moderate or severe pain symptoms, current smoking, and past-year cannabis, cocaine, other stimulant, and illicit opioid use), and time-varying covariates measured at first follow-up (past-year cannabis, cocaine, other stimulant, illicit opioid use, and unhealthy alcohol use).

### HIV subgroup analyses

AORs were similar between the HIV-stratified groups for depressive, anxiety, and pain symptoms, and illicit opioid use. AORs were larger in magnitude for individuals without HIV compared to with HIV for unhealthy alcohol, cannabis, and cocaine use, but CIs were very wide (Table 3).

**Table 3:** Odd ratios for each condition improving at second follow-up comparing quitters to non-quitters by HIV-status.

Condition improves	Odds ratios* (95% CIs)	
	HIV-negative	HIV-positive
Past-year unhealthy alcohol use	2.43 (0.88, 6.73)	1.25 (0.27, 5.76)
Past-year cannabis use	2.03 (0.66, 6.26)	1.29 (0.49, 3.44)
Past-year illicit opioid use	1.27 (0.48, 3.34)	0.98 (0.34, 2.88)
Past-year cocaine	9.68 (1.85, 50.6)	1.04 (0.42, 2.58)
Current depressive symptoms	1.06 (0.47, 2.40)	0.93 (0.26, 3.37)
Current anxiety symptoms	1.51 (0.71, 3.23)	0.68 (0.26, 1.79)
Current moderate or severe pain symptoms	1.06 (0.53, 2.09)	1.56 (0.63, 3.90)

*Adjusted for race, age, education, income, baseline conditions (AUDIT score, depressive symptoms, anxiety symptoms, moderate or severe pain symptoms, current smoking, and past-year cannabis, cocaine, other stimulant, and illicit opioid use), and time-varying covariates measured at first follow-up (past-year unhealthy alcohol, cannabis, cocaine, other stimulant, and illicit opioid use).

### Sensitivity analyses

While different assumptions about the causal structure of the data affected estimates, these differences were comparatively small and would not affect broader inferences for decision making. Assuming changes in smoking status between baseline and the next follow-up occurred *prior to* changes in use of cannabis, opioid, cocaine, and other stimulants reported at the next follow-up resulted in estimates that were generally larger than our primary estimates (Table 4, Fig 1A), ranging from 0.87 (0.50, 1.51) for improvement of depression to 2.95 (1.61, 5.41) for no longer using cocaine (Table 4, Fig 1B). In contrast, assuming changes in smoking status occurred *after* changes in depressive, anxiety, and pain symptoms resulted in estimates that were generally smaller than our primary estimates (Table 4, Fig 1A), ranging from 0.72 (0.40, 1.31) for improvement of depression to 2.18 (1.05, 4.53) for reduction in unhealthy alcohol use (Table 4, Fig 1C). Please see Fig 1 for visual representation of the different assumptions about the causal structure.

**Table 4:** Odd ratios for each condition improving at second follow-up under different assumptions about the causal structure.

Condition improves	Analysis	Odd ratios (95% CIs)
Past-year unhealthy alcohol use	Fig 1A *	2.13 (1.03, 4.39)
	Fig 1B **	2.85 (1.43, 5.68)
	Fig 1C ***	2.18 (1.05, 4.53)
Past-year cannabis use	Fig 1A *	1.67 (0.94, 2.97)
	Fig 1B **	2.17 (1.27, 3.72)
	Fig 1C ***	1.72 (0.98, 3.02)
Past-year illicit opioid use	Fig 1A *	1.09 (0.57, 2.07)
	Fig 1B **	1.28 (0.70, 2.33)
	Fig 1C ***	1.03 (0.54, 1.94)
Past-year cocaine use	Fig 1A *	2.12 (1.11, 4.03)
	Fig 1B **	2.95 (1.61, 5.41)
	Fig 1C ***	1.89 (0.99, 3.62)
Current depressive symptoms	Fig 1A *	0.74 (0.41, 1.34)
	Fig 1B **	0.87 (0.50, 1.51)
	Fig 1C ***	0.72 (0.40, 1.31)
Current anxiety symptoms	Fig 1A *	0.98 (0.60, 1.60)
	Fig 1B **	0.87 (0.50, 1.51)
	Fig 1C ***	0.72 (0.40, 1.31)
Current moderate or severe pain symptoms	Fig 1A *	1.24 (0.78, 1.95)
	Fig 1B **	1.30 (0.29, 2.01)
	Fig 1C ***	1.30 (0.82, 2.05)

*Primary weighted analysis reported in Table 2. Adjusted for HIV status, race, age, education, income, baseline conditions (AUDIT score, depressive symptoms, anxiety symptoms, moderate or severe pain symptoms, current smoking, and past-year cannabis, cocaine, other stimulant, and illicit opioid use), and *some* time-varying covariates measured at first follow-up (past-year unhealthy alcohol, cannabis, cocaine, other stimulant, and illicit opioid use).

**All time-varying covariates measured at first follow-up (past-year cannabis, cocaine, other stimulant, and illicit opioid use, unhealthy alcohol use, depressive, anxiety, and moderate or severe pain symptoms) *excluded* from the model for the weights.

***All time-varying covariates measured at first follow-up *included* in the model for the weights.

The E-values for the point estimates were 1.52 for depressive symptoms, 1.23 for anxiety symptoms, 1.55 for pain symptoms, and 2.26, 1.98, 1.28, and 2.37 for past-year use of unhealthy alcohol, cannabis, illicit opioids, and cocaine, respectively. The E-values’ lower limit CIs were 1.08 for unhealthy alcohol use, 1.42 for cocaine use, and 1.00 for the other outcomes.

## Discussion

Our study is the first attempt to emulate a hypothetical randomized trial of smoking cessation and to assess the impact of smoking cessation on co-occurring substance use, psychiatric symptoms, and pain symptoms among US veterans. We found evidence for greater discontinuation of co-occurring unhealthy alcohol, and cocaine after quitting smoking compared with not quitting smoking. The findings corroborate and strengthen the prior observational evidence indicating smoking cessation is significantly associated with reductions in some substance use [21, 22, 42, 43]. Though we additionally utilize observational data, the study advances knowledge by providing evidence based on a highly rigorous analysis approach emulating a randomized controlled trial by carefully controlling for selection and confounding bias. Determining whether smoking cessation is causally related to lower substance use has substantial population health importance, particularly for HIV-infected persons and disparity-impacted populations where their co-occurrence and prevalence are particularly high. Very high intensity smoking cessation interventions, including combinations of nicotine replacement therapy, pharmacotherapy, and talk therapy, may be warranted as first-line responses if smoking co-occurs with substance use. Conversely, smoking cessation interventions may be warranted as essential components of substance misuse treatments. Additional research to better understand the pathways linking tobacco reduction with reduction of other substance use is warranted.

We found substantial evidence that quitting smoking led to reductions in use of cocaine and alcohol. Our robust E-values for cocaine use (2.37) and unhealthy alcohol use (2.26) indicates that an unmeasured confounder would need to be strongly associated with both the exposure and the outcome to fully explain away our observed association, which strengthens our confidence in causal inferences. Implications may include viewing smoking cessation as a vital component of substance-misuse treatment for people using cocaine and/or with alcohol use disorders, or increasing the intensity of smoking cessation interventions, such as by combining talk therapy and pharmacotherapy with nicotine replacement therapy for smokers who also use cocaine and/or have alcohol use disorders.

We found no evidence that quitting smoking was associated with improvement of depressive, anxiety, and pain symptoms, and illicit opioid use in our sample of US veterans with and without HIV engaged in care in the VA. Our findings for depressive and anxiety symptoms are inconsistent with more recent findings that have found an improved impact of smoking cessation on psychiatric symptoms [23, 42]. This inconsistency may be a result of 1) methodological differences in this study compared to others (i.e. time of follow-up, lack of smoking relapse analysis in our study, categorization of depressive and anxiety symptoms) and/or 2) focusing on a specific high-risk population (HIV-infected and matched HIV-uninfected veterans). Still, the results corroborate more recent findings that smoking cessation does not exacerbate symptoms of depression and anxiety and overall worsen mental health.

PLHIV have greater risk for various mental and physical comorbidities in addition to conditions discussed in the current study [44, 45] and are more likely to engage in polypharmacy [46]. Therefore, this subpopulation may have unique differential effects of tobacco cessation on improvements in co-occurring conditions. When we stratified our analysis by HIV status to understand these effects, AORs for past-year unhealthy alcohol use, cannabis use, and cocaine use were somewhat larger for people without HIV compared with PLHIV, but CIs were wide with small subset of samples when stratified. Given higher prevalence of cigarette smoking and other co-occurring conditions in HIV populations [15–17], further research on understanding the impact of HIV status on smoking cessation and subsequent improvements in co-occurring conditions is warranted.

### Limitations

While the primary purpose of our analysis was to enhance the robustness of causal inference regarding the collateral benefit of smoking cessation, this analysis still falls short of a randomized trial because unmeasured and residual confounding cannot be ruled out. While we were able to measure and adjust for several potential indicators of quitting smoking, there are potential additional factors driving decisions to quit smoking that remained unmeasured, and some of our calculated E-values were quite small. Since information on cigarette smoking and other co-occurring conditions were assessed at each survey, the exact timing of smoking cessation and reduction of other co-occurring conditions was unknown. For example, if a current smoker quit smoking and reduced their unhealthy alcohol use between the first and second survey, it is unknown which event occurred first. However, we attempted to address this uncertainty with sensitivity analyses and found that all estimates were in the same direction and did not vary by more than approximately 25%. Further, we limited the exposure follow-up to the next survey (approximately one year post-baseline) to limit the variability in occurrence of covariates.

Even if the timing of smoking cessation was known, our results could still be biased by unmeasured confounding. The relatively small E-values for psychiatric symptoms, pain, and past-year cannabis and illicit opioid use indicate that there could be other factors like diagnosis of chronic diseases, other changes in health status, receipt of pharmacotherapy or other treatment, and financial or lifestyle changes that relate to smoking cessation and the resolution of these outcomes. Other limitations include the following: 1) the method of measurement for cigarette smoking and co-occurring conditions were assessed using brief screening tools or self-reported questionnaires that varied across time rather than clinical diagnoses, 2) our findings do not extend beyond the second follow-up (approximately two years post-baseline) and so we do not know if individuals maintained their smoking status and how this might affect outcomes over a longer period.

## Conclusion

While a causal interpretation of our findings may not be strictly warranted, our results expand the scope of causal inference regarding whether quitting smoking increases the likelihood of discontinuing unhealthy alcohol and cocaine use. Future studies are warranted to further clarify whether smoking cessation has causal inferences regarding substance use, such as by investigating sustained changes of co-occurring conditions over time and accounting for additional life events that may influence quitting smoking and changes in these co-occurring conditions. Overall, our findings support smoking cessation and its potential influence on discontinuing use of other substances.
